# A Multi-Locus Approach to Treating Fibromyalgia by Boosting Dopaminergic Activity in the Meso-Limbic System of the Brain

**DOI:** 10.4172/2157-7412.1000213

**Published:** 2014-01-27

**Authors:** Kenneth Blum, Marlene Oscar-Berman, Roger L Waite, Eric R Braverman, Florian Kreuk, Mona Li, Kristina Dushaj, Margaret A Madigan, Mary Hauser, Thomas Simpatico, Debmalya Barh

**Affiliations:** 1 Department of Psychiatry and McKnight Brain Institute, University of Florida College of Medicine, Gainesville, Florida, USA; 2 Departments of Psychiatry, Neurology, and Anatomy & Neurobiology, Boston University School of Medicine, and Boston VA Healthcare System, Boston, Massachusetts, USA; 3 Department of Nutrigeenomics, BioClarity, Inc. LaJolla, California, USA; 4 Department of Clinical Neurology, Path Foundation, NY, New York, New York, USA; 5 Department of Personalized Medicine, IGENE, LLC. Austin, Texas, USA; 6 Dominion Diagnostics, LLC, North Kingstown, Rhode Island, USA; 7 Human Integrated Services Unit University of Vermont Center for Clinical & Translational Science, College of Medicine, Burlington, Vermont, USA; 8 Institute of Integrative Omics & Applied Biotechnology, Nonakuri, Purba Medinipur, West Bengal, India; 9 Department of Addiction Research & Therapy, Malibu Beach Recovery Center, Malibu Beach, California, USA

Fibromyalgia (FM), is associated with fatigue, chronic diffuse pain, and cognitive/mood disturbances [1,2]. FM patients have greater healthcare costs and reduced workplace productivity. Stress and the neurotransmitter dopamine may interact, and this interaction may induce FM. Interestingly, FM has been called a ‘stress-related disorder’ presenting an exacerbation of symptoms on the context of stressful events [3].

Certainly, many genes are involved in FM causation and potential predisposition. However, it is important to realize that epigenetics may also be involved in the progression of this disorder. Specifically, Menzies *et al.* determined that FM was associated with acquired epigenetic/genetic changes [1]. Using gene-wide methylation analysis, Menzies *et al.* found that the majority of differentially methylated sites, as high as 91%, occurred in women with FM [1]. It is noteworthy, that these methylated sites involved nervous system, skeletal system, organ system, as well as chromatin compaction [1]. In particular, genes associated with methylate sites included *BDNF*, *PRKG1*, *NAT15*, *HDAC4*, *RTN1*, and *PRKCA* [1].

Moreover, Lukkahatai *et al.* found 107 genes that were differentially expressed between low and high pain groups of women with FM; furthermore, between low and high catastrophizing groupings of FM women, differential expression occurred in 139 genes [4]. In their exquisite study they identified interferon signaling and interferon regulatory activation factor pathways. It seems likely that interferon signaling may be able to distinguish between pain groups; however, between catastrophizing groups, dendritic cell maturation described it precisely [4].

In terms of genes that are linked to stress evocation, Light *et al*. from the University of Utah evaluated leukocyte gene expression resultant from resting and stressor-evoked changes [5]. They found support for physiological dysregulation and/or inherited susceptibility, especially for catechol-O-methyl transferase (*COMT*) genes (dopamine metabolism), the purinergic 2×4 (*P2X4*) ion channel, and the glucocorticoid and linked mineralocorticoid receptors (*NR3C1, NR3C2)* [5]. Involved in spinal microglia upregulation for persistent pain, these genes have been implicated as being involved in fatigue and muscle pain [5]. Furthermore, in a recent study by Smith *et al*. significant allele frequency differences between FM cases (496) and controls (348) was observed in three genes: *GBP1* (rs7911; P=1.06×10(−4)), *GABRB3* (rs4906902; P=3.65×10(−6)), and *TAAR1* (rs8192619; P=1.11×10(−5)) [6]. In addition, a meta-analysis performed by Lee *et al*. demonstrated that susceptibility to FM is conferred by the 5-HT2A receptor 102T/C [7]. Moreover, significant associations between DAT-1 and MAO-A polymorphisms and cold pain tolerance have been determined [8]. Differential tolerance was observed in carriers and homozygotes with certain alleles; for example, a shorter tolerance was observed in carriers of alleles 10 and 11, as compared to homozygous allele 9 [8]. Allele 4 carriers also had shorter tolerance compared with homozygous allele 3 [8].

The experience of pain is the result of both efferent and afferent systems, and pain is a main feature of FM. While stress-induced analgesia is known to be produced by acute stress, dependent on nucleus accumbens (NAc) dopamine-containing neurons, murine studies have shown that this response is eliminated by prolonged stress exposure, causing a stress-induced hypergesia state instead [9]. Within the NAc, dopaminergic activity has been shown to be attenuated by chronic stress; therefore, chronic stress has been hypothesized to play a role in stress-related hyperalgesia development.

Clinical studies have implicated dopaminergic function disruption, including decreased cerebrospinal fluid dopamine metabolites, as occurring in FM patients [10,11]. NAc dopamine release occurs in response to a variety of stressors, among them acute psychological stress, a major FM symptom [12]. A positive feedback loop occurs in FM patients, in which stress from pain exacerbates dopamine release causing hyperalgesia; the increased sensitivity to pain consequently results in further dopamine release. [12]. Following stress exposure to both chemical and thermal stimulants, hyperalgesia persists in rats for up to nine days [12]. Moreover, other neurotransmitters also play roles in this process. Clomipramine and fluextine, selective 5-HT reuptake inhibitors, and tryptaphan, a 5-HT reuptake precursor, have been shown to block hyperalgesia development, further suggesting that pain sensitivity duration and magnitude can be increased by repeated stress [12].

While both dopaminergic and serotonergic NAc functions are disrupted by chronic stress, the dopaminergic impact lasts longer than the impact on 5-HT [12]. In this regard, three possibilities have been proposed: (1) dopamine and 5-HT interact in a regulatory manner during stress-induced analgesia; (2) stress-induced analgesia inception is dependent on the disruption of this regulatory interaction; and (3) chronic stress-induced hyperalgesia may be caused by dopaminergic dysfunction, outlasting 5-HT, after normalization of serotonergic function [12].

This phenomenon potentially explains why chronic pain patient treatment strategies intended to increase only serotonergic function have had limited success in regards to analgesia [12]. Strategies intended to increase mesolimbic pathway dopaminergic function may be more effective since many reward type genes are associated with the stress-related FM disorder. In fact, a fast mirror to the brain and associated polymorphic genes which make a small but significant contribution, could be identified and incorporated into a panel of candidate reward genes tested to identify risk alleles as mentioned above, similar to those that are being developed in the addiction and pain field [13].

In summary, to our knowledge no one has attempted a combination therapy with numerous pharmacogenomics targets. TO The provision of a natural method of reward circuitry and signaling manipulation could be a viable solution. Utilizing a stress-reducing substance like Synaptamine^™^, a natural dopamine D2 agonist, impacting multi-locus neurotransmitters in the brain (meso-limbic and pre-frontal cortex and the cingulate gyrus) to stop the positive feedback loop in FM patients is warranted following required studies [14].

While there is a paucity of information linking the use of dopamine D2 agonist therapy and FM, we have shown earlier in mouse experiments that by utilizing DL-phenylalanine a known inhibitor of the enzyme enkephalinase, a significant increase in brain levels of enkephalin and a reduction of alcohol seeking [15] and others showed potentiation opioid induced analgesia [16].

We are hereby proposing that the coupling of a specialized genetic panel analyzing multi-loci reward gene polymorphisms [17] and the natural D2 receptor agonist [18] with minimal if any side effects-(similar ingredients have been utilized by 50,000 people to help them manage their cravings without any complaints filed with the FDA [19]) and enkephalinase inhibition [20] should provide an intelligent strategy to treat FM patients thereby attenuating inflammation and pain.

“The nutrients found in PhenCal 106 have been used safely by more than 50,000 people to help them manage their cravings without side effects,” said Luke Bucci, vice president of research for Weider Nutrition International, Inc. “We have not been contacted by any representatives of the FDA regarding PhenCal 106, nor have any complaints been filed.”
